# Demographic and Risk Factor Profile in Patients of Gallstone Disease in Central India

**DOI:** 10.7759/cureus.24993

**Published:** 2022-05-14

**Authors:** Aditya M Patel, Meenakshi Yeola, Chandrashekhar Mahakalkar

**Affiliations:** 1 Department of General Surgery, Datta Meghe Institute of Medical Science, Wardha, IND; 2 Department of General Surgery, All India Institute of Medical Sciences, Mangalagiri, Mangalagiri, IND

**Keywords:** demographic, gallstones, precaution, risk factors, cholelithiasis

## Abstract

Background

Gallbladder stones are more common in some regions of the world than others. Gallstones that are asymptomatic might be discovered as an afterthought during a regular ultrasound scan for another abdominal ailment. The changing incidence in India is mostly due to westernization and the availability of ultrasonography in both urban and rural areas, as well as increased affordability owing to changes in the socio-economic structure and the budget of investigations. This study aims to know the prevalence of gallstone disease as well as the risk factors that contribute to its development in central India.

Method

A single-center, cross-sectional observational study was conducted. Seventy-two radiologically diagnosed cases of gallstone disease irrespective of age, gender, and comorbid condition were included in the study.

Result

Seventy-two cases of gallstone were included in the research. The highest age-specific incidence of gallstone was in the fifth and sixth decades with the maximum incidence in the sixth decade. Females had a higher incidence of gallstone formation. The pain was the earliest symptom but we found that 41.67% patients had asymptomatic gallstones. A family history of gallstone disease is found positive in 69.44% of the patients who also had an increased risk of gallstone. 22.22% patient were only diabetic, 6.95% were only hypertensive and 20.83% were both diabetic + hypertensive. Comorbidity has a high prevalence of gallstone disease. Obesity has a significant link to gallstone disease, with BMI being one of the most important indicators of obesity.

Conclusion

The prevalence of asymptomatic gallstones is relatively high in central India. We strongly recommend ultrasonography as a screening modality in patients with older age group, female gender, high cholesterol level, family history of gallstones, sickle cell disease, increased BMI and co-morbidities like diabetes or hypertension for early detection of gallstones formation.

## Introduction

Gallbladder stones are more common in some regions of the world than others. In India, it is considered to be approximately 4%, whereas it is 10% in the Western world [1].

Gallstones are frequently discovered by accident on ultrasonography, computed tomography scans, abdominal radiography, or laparotomy in individuals who have no biliary symptoms. Every year, around 3% of those who are asymptomatic become symptomatic. Over the course of a 20 years span, nearly two-thirds of asymptomatic gallstone patients stay symptom-free [2].

Gallstones are diagnosed by combining a thorough history and physical examination with the appropriate investigation. The changing incidence in India is mostly due to westernization and the availability of ultrasonography in both urban and rural areas, as well as increased affordability owing to changes in the socio-economic structure and the budget of investigations.

Because of the rising occurrence of gallstone and its numerous manifestations in India, research that can offer information on the disease's prevalence, various clinical presentations, therapy, and outcomes is urgently needed.

This study aims to know the prevalence of gallstone disease as well as the risk factors that contribute to its development in central India. It also aims to uncover significant epidemiological factors to better understand the pathogenesis of gallstone disease and to develop effective therapy and preventative methods.

## Materials and methods

The present cross-sectional observational hospital-based study was conducted in the Department of Surgery at Acharya Vinoba Bhave Rural Hospital (AVBRH), a tertiary level hospital, situated in the rural area of Wardha district, in central India, attached to Jawaharlal Nehru Medical College, Sawangi (Meghe), Wardha, Maharashtra, and affiliated to the Datta Meghe Institute of Medical Sciences, DMIMS (DU), Wardha from October 2019 to October 2021 after approval from Institutional Ethical Committee (IEC). Seventy-two radiologically diagnosed cases of gallstone disease irrespective of age, gender, and co-morbid conditions were included and gallstone diseases with gallbladder malignancy were excluded in the study. Detailed history, past history, personal history including the history of any addictions, and investigations reports were obtained.

Statistical tools

The presentation of the categorical variables was done in the form of numbers and percentage (%). The quantitative data, on the other hand, were presented as means ± standard deviations (SD) and as a median with 25th and 75th percentiles (interquartile range). The data were entered into a Microsoft EXCEL spreadsheet, and the final analysis was carried out utilizing IBM's Statistical Package for Social Sciences (SPSS) software, version 21.0 (IBM, Chicago, IL, USA).

## Results

In the present study, 23.61% of patients belonged to the age group 61-70 years, followed by 51-60 years (18.06%), 41-50 years (16.67%), 21-30 years (12.50%), 31-40 years (12.50%), and =20 years (11.11%). The age group was > 70 years old for only four out of 72 patients (5.56%). The mean age (years) of study subjects was 47.11 19.1, with a median (25th-75th percentile) of 50 (34.25-63) (Table 1).

**Table 1:** Age (years) distribution of study subjects.

Age(years)	Frequency	Percentage
<=20	8	11.11%
21-30	9	12.50%
31-40	9	12.50%
41-50	12	16.67%
51-60	13	18.06%
61-70	17	23.61%
>70	4	5.56%
Mean ± SD	47.11 ± 19.1	
Median(25th-75th percentile)	50(34.25-63)	
Range	5-82	

Of total 72 subjects, 40 patients were females and 32 patients were males. The mean value of weight(kg) of study subjects was 62.18 ± 18.7 with a median (IQR) of 60(50-76). In the present study, 44.5% of patients had normal BMI, followed by overweight patients 41.6%. Ten patients were obese out of 72 patients (13.9%) (Table 2).

**Table 2:** Distribution of BMI of study subjects.

BMI	Frequency	Percentage
Normal	32	44.5%
Over weight	30	41.6%
Obese	10	13.9%
Total	72	100.00%

The majority (51.39%) of patients experienced dull aching pain, followed by asymptomatic (41.67%). Colicky pain was present in only five out of 72 patients (6.94%). Similar history was present in only 24 out of 72 patients (33.33%). Family history was present in only 50 out of 72 patients (69.44%). 50% of patients had no co-morbidities, followed by diabetes (22.22%) and hypertension (6.95%). Fifteen patients (20.83%) had both diabetes and hypertension (Table 3).

**Table 3:** Distribution of comorbidities of study subjects.

Co-morbidities	Frequency	Percentage
No co-morbidities	36	50%
Diabetes	16	22.22%
Hypertension	5	6.95%
Diabetes + hypertension	15	20.83%
Total	72	100%

Out of 72, the majority (86.11%) of patients had a mixed diet. The diet was vegetarian in only 10 out of 72 patients (13.89%). The majority (55.56%) of patients did not have any addiction, and 30.56% of patients were tobacco consumers. Only 10 out of 72 patients (13.89%). Five percent of women were OCP users. Only one out of 40 women (2.5%) was pregnant.

In the present study, in the majority (56.94%) of patients, random blood sugar (mg%) was normal (70 to 150 mg%). Random blood sugar (mg%) was deranged in only 31 out of 72 patients (43.06%). Mean value of random blood sugar (mg%) of study subjects was 153.46 ± 77.12 with median (25th-75th percentile) of 109.5 (90-202.5). In the majority (56.94%) of patients, HbA1C was <6% (non diabetic) followed by >7% (action suggested) (30.56%). HbA1C was 6% to 7% in only nine out of 72 patients (12.5%). Mean value of HbA1C (%) of study subjects was 6.1 ± 1.32 with median (25th-75th percentile) of 5.35 (5-7.125).

In present study, in the majority (61.11%) of patients, total cholesterol (mg/dL) level was desirable (<200 mg/dL) followed by high {>240 mg/dL} (20.83%). Total cholesterol (mg/dL) level was borderline (200 to 239 mg/dL) in only 13 out of 72 patients (18.06%). Mean value of total cholesterol (mg/dL) levels of study subjects was 188.19 ± 70.91 with median (25th-75th percentile) of 155 (132.75-235.25) (Table 4).

**Table 4:** Distribution of total cholesterol (mg/dL) levels of study subjects.

Total cholesterol (mg/dL) levels	Frequency	Percentage
Desirable {<200 mg/dL}	44	61.11%
Borderline {200 to 239 mg/dL}	13	18.06%
High {>240 mg/dL}	15	20.83%
Mean ± SD	188.19 ± 70.91	
Median (25th-75th percentile)	155(132.75-235.25)	
Range	100-354	

In the present study, in the majority (93.06%) of patients, ultrasonography abdomen + pelvis finding was cholelithiasis followed by cholelithiasis with cholecystitis (4.17%). Ultrasonography abdomen + pelvis finding was cholelithiasis with choledocholithiasis in only two out of 72 patients (2.78%) (Table 5).

**Table 5:** Distribution of abdominopelvic ultrasonography of study subjects.

Ultrasonography abdomen + pelvis	Frequency	Percentage
Cholelithiasis	67	93.06%
Cholelithiasis with choledocholithiasis	2	2.78%
Cholelithiasis with acute cholecystitis	3	4.17%
Total	72	100.00%

## Discussion

The present hospital-based study was conducted in the Department of Surgery at Acharya Vinoba Bhave Rural Hospital (AVBRH), a tertiary level hospital, situated in the rural area of Wardha district, in central India, attached to Jawaharlal Nehru Medical College, Sawangi (Meghe), Wardha, Maharashtra, and affiliated to the Datta Meghe Institute of Medical Sciences, DMIMS (DU), Wardha from October 2019 to October 2021.

In this study, the results of our study were compared with those of well-known authors. In total, 72 radiologically diagnosed cases of gallstone disease irrespective of age, gender, and co-morbid conditions were included in the study. Following the detailed history, past history, and personal history, including a history of any addictions, and investigation reports, the following observations were noted.

Age

The age of the participants in this research ranges from 11 to 82 years. The fifth and sixth decades have a higher incidence, with the sixth decade having the highest incidence. Herman et al. found a similar frequency in their research (sixth decade) [3]. In the fourth decade, Bhagavan's series reached its peak [4]. 

Gallstones become more likely as you become older [5]. Bile acid production declines with age, biliary cholesterol production rises, and cholesterol saturation rises. These side effects are caused by a decrease in the action of cholesterol 7a-hydroxylase (CYP7A1), the bile acid production rate-limiting enzyme [6,7]. Furthermore, as people become older, more risk factors might accumulate and cause lithogenesis [8].

Sex

In this study, 40 of the 72 cases were female, with the remaining 32 being male. Several authors [9-12] have confirmed that females had a greater prevalence of cholelithiasis than males, as seen in our study. Maternity and sex hormones are thought to put women at greater risk, and some traditional epidemiologic studies back this up. Estrogen enhances biliary cholesterol release, leading bile to become cholesterol hyper-saturated and lithogenic [11].

Character of pain

The commonest site of pain was the right hypochondrium and the next commonest site was the epigastrium. In present study, 37 patients out of 72 had dull aching pain, five patients had an acute onset of pain which was colicky in nature and remaining 30 patients were asymptomatic who were screened with ultrasonography and incidentally diagnosed with gallstone disease. A similar higher incidence of asymptomatic cholelithiasis was noted in Sachdeva et al.'s study [11]. We also found that 33.33% (24 patients out of 72) had similar attack of pain occurred previously before diagnosed with cholelithiasis.

Family history of gallstones disease

Fifty out of 72 patients (69.44%) had a family history of gallstone disease. A similar incidence of cholelithiasis was found with a positive family history of gallstone disease in the study done by Sodhi et al. [13] and Sachdeva et al. [11].

Jackson and Gay published the first investigation into the link between gallbladder disease and family history in 1959 [14]. Since then, several studies of gallstone in first-degree relatives of gallstone patients have indicated that first-degree families of gallstone patients are two to four considerably more probable than stone-free individuals to develop gallstone [15]. Furthermore, siblings of gallstone patients may acquire lithogenic bile at a young age as a consequence. Even if family members have similar lifestyles, such as dietary habits, genetic predisposition plays a role in gallstone formation [16]. The hereditary vulnerability to gallstone disease, as well as shared lifestyle and metabolic variables, might be the underlying mechanism. The genetic and other processes involved in the development of biliary stones can be clarified by epidemiologic investigations that use genomic and metabolic techniques [17].

Comorbidities 

Sixteen out of 72 patients had only diabetes, five patients had only hypertension, and 15 patients had both diabetes and hypertension as a co-morbidity. We found positive comparison with compared our data with Sachdeva et al. [11] and Gupta et al. [18].

We conducted the study in rural areas where the patients were not aware of cholelithiasis. Here we screened a patient who had risk factors for cholelithiasis with ultrasonography. We found 41.60% having asymptomatic gallstones and among them, 43.06% having diabetes, which is suggestive of the risk factor for causing gallstone disease. Epidemiological studies have found a link between diabetes and gallstone disease. Gallstone disease has been discovered to be more common among diabetics [13]. After controlling for age, BMI, waist/hip ratio, and ethnicity, the self-reported prevalence of gallstone disease was 1.6 times greater in women with diabetes than in women without diabetes in population-based research [19]. Fasting gallbladder volume in diabetic patients is higher than in controls, indicating gallbladder hypotonicity and bile stasis. Both after a meal and after an infusion of cholecystokinin, diabetic patients' bile ejection fraction is lowered following stimuli [20]. Reduced gallbladder motility may also be a result of diabetic neuropathy [21].

Comorbid diseases such as hypertension, diabetes, and hypercholesterolemia were often detected in patients, however in univariate analysis, only hypertension was found to be substantially linked with gallstones [11]. The current study's finding of a link between gallstones and recent stress (physical and/or psychological) might be due to the well-known link between stress and hypertension.

Obesity

In our study of 72 patients, 32 (44.5%) patients had BMI within normal limits (<25kg/m^2^) and 40 (55.5%) had BMI above normal limits (>25kg/m^2^). The similar studies done by Sarhan et al. [22] and Sodhi et al. [13] are very significant suggesting raised BMI is involved in the pathogenesis of gallstone disease.

Because of the link between obesity and gallstones, previous research used the body mass index (BMI) as a common approach for calculating the risk of acquiring gallstones. The arrangement of fat in the body, in relation to overall body fat, is a potential risk for gallstone development. Abdominal obesity is connected to a range of metabolic diseases in both female and male patients, including the development of gallstones. Increased BMI appears to be the most prominent preventable risk factor in this community, and hence appears to account for the region's high gallstone prevalence.

In the present study, 61.11% of patients, total cholesterol was desirable followed by high in 20.83% and borderline in 18.06%. So, 38.89% patients had hypercholesterolemia in this study. In the study conducted by Alexander et al. [23] and Hayat et al. [24] had hypercholesterolemia which is comparable to our study. High serum cholesterol concentrations were independently associated with the risk of gallstones. A small number of scientists have proposed that some mental stressors are linked to an increase in blood cholesterol levels, and that hyper cholesterol predisposes to gallstone development [25].

Obstetric history

In the present study, six women were noted as an oral contraceptive pill user and only one female was pregnant while diagnosed with gallstone disease. In the study conducted by Sachdeva et al., 11 women out of 91 were found to oral contraceptive user and no pregnant lady diagnosed with gallstone disease [11]. Similar study was conducted in 1991 by Vecchia in Milan, Italy. Who also noted only 48 women out of 235 who used oral contraceptive pills and developed cholelithiasis [26].

Contraception was not shown to be a major risk factor for gallstone disease in our investigation. This conclusion was consistent with previous researches by Sachdeva et al. [11], Caroli-Bosc et al. [27], and by Vecchia in Milan [26] but not with another earlier study by Khan et al. [28], which found that female contraceptive usage is a meaningful risk factor for gallstones. The effect of contraception on gallstone development was linked to estrogen content, which might have a dose-dependent effect [29]. Low-dose estrogen in some kinds of contraception that have recently been used may provide little or no danger, and this idea might explain our findings of a negative link between contraception and gallstones.

Dietary habit

Sarda et al. stated that among the patients consuming vegetarian and non-vegetarian diets, gallstones were found to be more common, accounting for 80%of the cases with mixed dietary habits [30]. As far as dietary habits were concerned, 13.89% of our patients were vegetarians and 86.11% consumed mixed diet. This shows that cholelithiasis is more common in the patients consuming non vegetarian diet. 

Tobacco Consumption

30.56% of patients in this study were addicted to Kharra consumption which is a commercially manufactured kind of smokeless tobacco. Kharra is eaten by softly sucking and chewing a pinch of it between the gum and cheek, similar to chewing tobacco. The Kharra begins to melt and turn a rich red hue within minutes of chewing and mingling with saliva. It may provide a “buzz” that is slightly stronger than tobacco chewing, snuffing, or smoking. The usage of smokeless tobacco is very addicting. Smokeless tobacco's nicotine is more quickly absorbed than that found in cigarettes, making it more addictive.

Nicotine reduces mucin formation and secretion in the gallbladder by reducing prostaglandin synthesis, and has been linked to increased cholesterol precipitation and crystallisation in the bile [31]. Other researchers, on the other hand, discovered no link between tobacco use and the occurrence of gallbladder stones, and even detected a preventive effect against the production of gallbladder stones [12,32,33]. Nicotine's dose-dependent impact might be one explanation for these inconsistent results. Another reason for these inconsistent studies is the difficulty of evaluating nicotine usage subjectively and quantitatively. Depending on the nicotine intake, studies reveal an increase in bile acids [34].

Alcohol Consumption

Ten (13.89%) patients out of 72 were chronic alcoholic. The majority of research found that moderate alcohol intake had a protective impact on gallbladder stone prevalence [35-37]. The impact of alcohol causing a rise in the HDL fraction and the related decline in the cholesterol saturation index might be one reason for the preventive effect of moderate alcohol use in the genesis of gallbladder stone disease [31]. Excessive alcohol use, on the other hand, is viewed as a factor that promotes gallbladder stone development due to the subsequent liver damage [38]. A reduction in the amounts of apolipoproteins AI and AII may be one cause for the higher occurrence of gallbladder stones in those who consume a lot of alcohol and have liver impairment [39].

Sickle cell disease

Tripathy noticed eight patients out of 80 patients of sickle cell disease in Burla, Orissa [40]. Similarly, we found six patient having sickle cell disease out of 72 patients of gallstone disease. Pigment gallstones are more common in people with chronic hemolytic anemias, such as sickle cell anemia and thalassemia. Gallstone development is caused by an increase in unconjugated bilirubin excretion, bilirubin precipitation, and the creation of bilirubinate crystals.

This study was done in the Vidarbha region of Maharashtra state in India, where prevalence of sickle cell disease is increasing. So, it is important to keep in mind the possibility of the formation of gallstones in known cases of sickle cell disease while treating them.

Cirrhosis of liver

In our study, 10 (13.89%) patients out of 72 were chronic alcoholic so their liver had mild to moderate cirrhotic changes noticed through radiological modality.

The most prevalent cause of pigment lithogenesis in adults is liver cirrhosis. Cholesterol stones are seen in just a tiny percentage of cirrhotic individuals. Modifications in bile constituents (supersaturation of the bile in calcium bilirubinate for pigment stones or supersaturation in cholesterol for cholesterol stones), enhanced crystal nucleation in the presence of mucin and its congeners, and gallbladder hypomotility (stasis) that allows crystals to grow into gallstones are the major abnormalities that lead to gallstone formation [41]. Gallstones are frequently asymptomatic in people with liver cirrhosis, and they have a higher likelihood of being discovered by ultrasonography during routine liver disease check-ups.

Ultrasound findings

Ultrasound scanning was done on all the patient and all the cases revealed stone in the gallbladder. Two patients had additional stone in their common bile duct also and three patients had cholecystitis along with gallstone.

In cases of suspected gallbladder or biliary illness, ultrasonography is the method of choice. It is a very sensitive, specific, noninvasive, and cost-effective test for detecting gallstones. Furthermore, it is easy, quick, and safe during pregnancy, and the patient is not exposed to hazardous radiation or I.V. contrast. It has the added benefit of being able to be conducted at the bedside by a qualified practitioner. When it comes to identifying simple acute cholecystitis, ultrasound is really helpful. Gallbladder wall thickening (> 3mm), pericholecystic fluid, and sonographic Murphy's sign (probe tenderness over the visualized gallbladder) are all sonographic features of acute cholecystitis [18].

## Conclusions

The prevalence of asymptomatic gallstones is relatively high in the central India. Female gender, age, high cholesterol level, family history of gallstones, sickle cell disease and increased BMI are independent risk factors. The prevalence of asymptomatic gallstones is relatively high in the central India. Understanding the pathophysiology of gallstone disease can aid nurses in providing resources and information to patients who have been diagnosed with gallstones, as well as cholelithiasis prevention.

We strongly recommend ultrasonography as a screening modality in patients with older age group, female gender, high cholesterol level, family history of gallstones, sickle cell disease, increased BMI and co-morbidities like diabetes or hypertension for early detection of gallstones formation.
